# Experiences of Gender Inequity Among Women Physicians Across Career Stages: Findings from Participant Focus Groups

**DOI:** 10.1089/whr.2021.0051

**Published:** 2022-03-28

**Authors:** Sherry S. Chesak, Manisha Salinas, Helayna Abraham, Courtney E. Harris, Elise C. Carey, Tejinder Khalsa, Karen F. Mauck, Molly Feely, Lauren Licatino, Susan Moeschler, Anjali Bhagra

**Affiliations:** ^1^ Department of Nursing, Division of Nursing Research, Mayo Clinic, Rochester, MN, USA.; ^2^ Department of Medicine, Division of General Internal Medicine, Mayo Clinic, Jacksonville, Florida.; ^3^ Department of Medicine, Mayo Clinic, Rochester, MN, USA.; ^4^ Department of Medicine, Division of Infectious Disease, Massachusetts General Hospital, Boston, Massachusetts, USA.; ^5^ Department of Medicine, Division of Community Internal Medicine, Geriatrics and Palliative Care, Mayo Clinic, Rochester, MN, USA.; ^6^ Department of Medicine, Division of General Internal Medicine, Mayo Clinic, Rochester, MN, USA.; ^7^ Department of Anesthesiology and Perioperative Medicine, Central Division Mayo Clinic, Rochester, Minnesota, USA.; ^8^ Department of Anesthesiology, Division of Pain Medicine, Mayo Clinic, Rochester, Minnesota, USA.

**Keywords:** women physicians, gender, medicine, inequity, challenges, bias

## Abstract

**Background and Purpose:**

Gender inequity in academic medicine persists despite efforts to the contrary. Even with increasing representation of women physicians in academic medicine, leadership positions and promotion to tenure are still not representative. This study describes the experiences of women physicians at various stages of their careers, uncovering current challenges and potential areas for improvement toward gender equity.

**Methods:**

Three focus groups were conducted (*n* = 28) as part of a national professional development conference: Growth, Resilience, Inspiration, and Tenacity (GRIT) for Women in Medicine: GRIT. We thematically analyzed participant responses to assess perspectives on the impact of experiences, barriers to professional growth, opportunities for improvement, and definitions of success.

**Results:**

The major issues the participants faced included subthemes of (1) systemic barriers to success, (2) implicit biases, (3) self-advocacy, and (4) burnout and stress. Solutions for issues that were discussed included (1) fostering supportive communities, (2) encouraging personal and professional development, and (3) the need for system-wide policy changes. We found that most women needed or benefited from the fostering of communities and desired opportunities for developing professional skills. Participants felt institutional transparency for grievances determined the level of support and confidence in reporting instances of mistreatment. Participants tended to define success according to (1) personal success and (2) leaving a legacy.

**Conclusions/Implications:**

Despite policy advancements and a social evolution away from discrimination against women, women in medicine continue to experience inequities across career stages. Potential solutions include fostering supportive communities, encouraging personal and professional development, and system-wide policy changes.

## Introduction

There has been increased recognition of gender disparities in academic medicine internationally.^1^ The number of women in medicine has grown consistently over 50 years such that 50% of medical school graduates^2^ and 36% of physicians^3^ in the workforce are women. Despite this growth in numbers, the percentages of those in leadership positions in organized medicine and academia have consistently remained low.^4^ In fact, in a 2021 study, it was identified that only 13% of hospital system leaders are female, 14% are chief medical officers and 27% are chief executive officers.^5^ In academic medicine, women comprise only 20% of department chairs of medical schools and 18% of dean positions.^6^ Given that this gender inequity has persisted for decades, immediate action is warranted.^7,8^

### Barriers to women's advancement in academic medicine

The challenges women face in academic medicine are complex and deeply rooted in persistent structural and systemic inequalities.^9^ Women physicians face barriers to advancement at all stages of their career related to gender inequities,^10–12^ disproportionate burden of domestic and parental responsibilities,^13–15^ work-life integration challenges,^14,16^ and threats to their career attributed to pregnancy, child birth, and child rearing.^4,12,17,18^ Significant barriers to the advancement of female medical faculty are heightened by established bureaucratic and authoritarian structures.^9,11^

Gender-based norms, behaviors, and expectations in medical institutions are dauntingly complex and vary throughout the progression of a career.^9^ Women are less likely to have a voice in hiring practices, and are often seen as less capable or qualified for leadership opportunities.^19^ In addition, female faculty encounter more barriers than male faculty, such as less administrative support, less satisfaction with careers, fewer publications and funded grants, and stalled or slower career progression.^7^

Furthermore, many academic medical centers do not have formal institutional policies to promote recruitment, promotion, and retention of female faculty, instead focusing on individual programs, but lacking systematic plans focusing on gender equity and career advancement of women.^20^ While there has been an upward movement toward increasing workforce diversity, the lack of inclusive practices continues to be a structural challenge faced by women. Ideally, inclusion for women in the workplace would involve equitable opportunities for professional growth, freedom from stereotyping, fair working conditions and policies, and support for work-life integration.^21^

### Benefits to women in leadership positions in medicine

When women are in leadership positions in medicine, there are several benefits to the institutional workforce, as well as personal and professional development. Women in leadership positions can create a positive and powerful impact on diversity and inclusion of rising physicians as representative role models.^7^ Peer or other types of mentoring, effective networking, and targeted programs geared for enhancing professional development have demonstrated positive outcomes and increased sense of self-reported skills and capabilities.^19^ When there is demonstrated advocacy for women's leadership on medical campuses and opportunities to gain skills and develop collegial networks, motivation, perception of success, and confidence for success in careers can be greatly improved.^22^

Leadership development programs have been shown to be especially impactful in positively shaping careers by building networks and skills, and promoting the cultivation of interpersonal and professional relationships.^22^ In a landmark study reporting the outcomes of an intervention to correct gender-based obstacles to career advancement for women at Johns Hopkins University School of Medicine, the findings indicated that women physicians benefited with retention and promotion of qualified women faculty, salary equity, quality of mentoring and integration into the scientific community, and decreased gender bias.^23^ However, interestingly, men physicians also reported improvements in the same areas.^23^

In addition, research indicates that an increase in women in top business leadership positions results in a number of benefits to the organization, including improvements in the firm's value, financial performance, economic growth, innovation, insolvency risk, social responsiveness, philanthropy, and more stringent monitoring and oversight and fewer legal infractions.^24^ Rationale for these improvements have been attributed to an increase in homogeneity of ideas and perspectives; in addition, women tend to exhibit transformational leadership styles that foster employee morale, motivation, and performance.^25^

### Previous research

Previous studies examining women physicians and leadership have found that including women at all levels of the conversation—planning initiatives, policies, and evaluating practices—is essential to promoting inclusion and equity in academic medicine leadership.^26^ Examining narratives of women in academic medicine can reveal insight on strategies, which promote more equitable practices and inclusive climates.^22^

Previous qualitative research has also suggested generational differences in attitudes, beliefs, and behaviors toward gender parity in academic medicine;^7,22,27^ however, few studies have investigated the barriers and supportive factors associated with the professional development of women in academic medicine based on the various stages of their careers. The purpose of this study is to assess perceived challenges encountered by women physicians at varying stages of their career, and to identify coping strategies for overcoming challenges and achieving professional success.

## Methodology

### Study design

This qualitative descriptive study is phase II of a two-phase study. Phase I entailed quantitative methods to assess demographics of physicians who attended Growth, Resilience, Inspiration, and Tenacity (GRIT) for Women in Medicine: GRIT, a national conference and continuing medical education course, to ascertain their perceptions of gender discrimination and disparity in the workplace, and potential solutions to address those issues. Outcomes from the phase I study are reported elsewhere.^28^

The current phase II study involves focus group interviews with conference attendees to (1) develop an accurate account of physician women's lived experiences with gender discrimination and disparity, (2) provide a description of coping strategies they have employed in an attempt to overcome the related challenges, and (3) identify how they defined success in their careers.

### Sample and sampling method

Participants included a convenience sample of attendees at the GRIT conference. The advertised key aim of the course was to “empower women and men in medicine with the skills and resources to remove barriers and bias of women in leadership positions specific to the challenges in healthcare.” To be included in the study, participants were required to attend the course in-person, identify as female physicians, and read and communicate in English.

All attendees at the conference were verbally informed of the study purpose during the opening session of the course and were invited to participate. Sign-up sheets were provided for three focus groups with 10 openings in each; once each sign-up sheet was filled, recruitment was ended. Two individuals who signed up did not participate, with no reason provided; therefore, the total number of participants was 28.

### Focus group structure and data collection

The three focus groups were composed of participants at varying stages of their careers. Focus group 1 included early-career physicians (those in their first 10 years of practice); group 2 included mid-career physicians (11–20 years of practice); and group 3 comprised late-career physicians (>20 years of practice). The interviews were all conducted at the same time at the location of the conference; thus a different investigator (T.K., L.L., S.S.C.) facilitated each one and each was joined by an assistant moderator (M.F., E.C., K.M.) who took field notes during the interviews. All interviewers and moderators were female and had expertise in conducting focus group interviews.

The interviews were each 1 h in length and an interview guide (Table 1) was used to ensure consistency in data collected across the three groups; the questions were developed according to guidelines for minimizing bias and enhance the reliability and validity of interview data.^29^ The interviews were recorded and transcribed verbatim by a professional transcriptionist. No identifying information was included in the transcripts.

**Table 1.** Interview Guide for Semi-Structured Focus Groups

Opener: What comes to the forefront of your mind when you think about being a woman in medicine? (thoughts/feelings/attitudes) Can you think of a time when gender seemed to play a role in your interactions at work? Were there any unspoken factors in your work environment? What kinds of issues did that raise, if any? Is this a symptom of a larger problem? Did anyone else have an experience of feeling overlooked or limited in this way? Did that encounter have downstream effects? What were your solutions at that time? Looking back, would you have done it differently? Was your outward reaction different than your inward reaction? When you think of being successful in your career, what does that look like now? When it comes time to finish your career in medicine, what does that success look like?

### Data analysis

Data were analyzed using a descriptive qualitative approach as described,^30^ and a thematic analysis^31^ was conducted to identify and analyze patterns or themes in the data according to frequency, specificity, amount of emotion, and extensiveness of the data.^32^

First, the transcripts were read in entirety by eight investigators and each independently identified emerging themes. Then the investigators compared and contrasted themes across the analyses and collaboratively integrated them into one structure of themes. Next, four investigators (S.S.C., C.H., M.S., and H.A.) reread all transcripts and assigned statements to appropriate themes according to the new thematic structure. Once again, analyses were compared and contrasted, and a final assignment of themes was developed for all statements. The three main themes were identified in advance according to the purpose of the interviews, while the subthemes were derived from the data.

### Ethical considerations

This study was approved by the Mayo Clinic IRB, and all participants signed a consent form before participation. The standards of the United States federal policy for the protection of human subjects were followed by the investigators.

## Results

Twenty-eight individuals agreed to participate, and all identified as female per the inclusion criteria (other demographic data reported in Table 2). During the analysis process, common subthemes were identified throughout the discussions, many of which were identified across the groups (early career, mid-career, and late career) and other subthemes that differed between the groups (Table 3).

**Table 2.** Demographic Data

Demographic	Group 1 early career (*n* = 10)	Group 2 mid career (*n* = 8)	Group 3 late career (*n* = 10)	Total (*n* = 28)
Age				
25–35 years	3 (30%)	0 (0.0%)	0 (0.0%)	3 (10.7%)
36–44 years	6 (60%)	2 (25%)	0 (0.0%)	8 (28.5%)
45–54 years	0 (0.0%)	5 (62.5%)	2 (20%)	7 (25%)
55–64 years	0 (0.0%)	0 (0.0%)	6 (60%)	6 (21.4%)
65 years or older	0 (0.0%)	0 (0.0%)	0 (0.0%)	0 (0.0%)
Missing	1	1	2	4
Race				
Native American/Alaskan Native	0 (0.0%)	0 (0.0%)	0 (0.0%)	0 (0.0%)
African American	0 (0.0%)	1 (12.5%)	0 (0.0%)	1 (3.57%)
Hispanic/Latino	1 (10%)	0 (0.0%)	1 (10%)	2 (7.14%)
Asian/South Asian	1 (10%)	2 (25%)	1 (10%)	4 (14.2%)
White	7 (70%)	4 (50%)	6 (60%)	17 (60.7%)
Multiracial	0 (0.0%)	0 (0.0%)	0 (0.0%)	0 (0.0%)
Other	0 (0.0%)	0 (0.0%)	0 (0.0%)	0 (0.0%)
Missing	1	1	2	4
Partner status				
Married/committed partnership	8 (80%)	5 (62.5%)	7 (70%)	20 (71.4%)
Single	1 (10%)	2 (25%)	1 (10%)	4 (14%)
Missing	1	1	2	4
Partner works outside the home				
Yes, full time	6 (60%)	5 (62.5%)	5 (50%)	16 (57.1%)
Yes, part-time	1 (10%)	0 (0.0%)	1 (10%)	2 (7.1%)
No	1 (10%)	0 (0.0%)	1 (10%)	2 (7.1%)
Missing	2	3	3	8
Partner is also a physician				
Yes	1 (10%)	1 (12.5%)	3 (30%)	5 (17.9%)
No	5 (50%)	4 (50%)	3 (30%)	12 (42.9%)
Missing	4	3	4	11
Number of children (including stepchildren)				
0	3 (30%)	1 (12.5%)	0 (0.0%)	4 (14.3%)
1	2 (20%)	1 (12.5%)	2 (20%)	5 (17.9%)
2	2 (20%)	5 (62.5%)	3 (30%)	10 (35.7%)
3	2 (20%)	0 (0.0%)	2 (20%)	4 (14.3%)
4	0 (0.0%)	0 (0.0%)	1 (10%)	1 (3.6%)
Missing	1	1	2	4
Current geographic location in the US				
Northwest	0 (0.0%)	1 (12.5%)	0 (0.0%)	1 (3.6%)
Northeast	1 (10%)	0 (0.0%)	0 (0.0%)	1 (3.6%)
Midwest	3 (30%)	3 (37.5%)	3 (30%)	9 (32.1%)
South	3 (30%)	1 (12.5%)	2 (20%)	6 (21.4%)
West	2 (20%)	2 (25%)	4 (40%)	8 (28.6%)
Missing	1	1	1	3
Mayo Clinic employee (results for conference at large)				
Yes				30.1%
No				69.9%

**Table 3.** Focus Group Data Themes with Number of Statements per Career Stage Group

Themes and subthemes	Number of statements		
	Group 1 early career	Group 2 mid-career	Group 3 late career
1. Issues women in medicine face			
1A. Systemic barriers to success	1	15	25
1B. Implicit biases	11	11	10
1C. Self-advocacy	6	12	9
1D. Burnout/stress	0	4	1
2. Solutions for issues women in medicine face			
2A. Fostering supportive communities	20	24	8
2B. Encouraging personal/professional development	19	13	9
2C. System wide policy changes	5	12	16
3. Defining success			
3A. Personal success	11	13	6
3B. Leaving a legacy	13	7	14

### Major issues faced by women in medicine

Participant responses indicated many unique obstacles faced by women in medicine. They identified four main subthemes: (1) systemic barriers to success, (2) implicit biases, (3) self-advocacy, and (4) burnout/stress.

### Systemic barriers to success

The participants, especially in the mid- and late-career groups, identified many systemic barriers to success, which included policies and practices resulting in inequity for female physicians. Systemic inequities commonly described by the participants included maternity policies, unequal compensation, and sexual harassment. A woman in the mid-career group reported her pregnancy before joining a private practice group. After disclosing, she stated that she was informed that the offer was no longer on the table, as the group could not “invest in that.” Other women in the same group reflected on similar experiences.

The late-career group discussed issues surrounding sexual harassment. In their experiences regarding sexual harassment complaints, the complainant often resigns while the harasser does not face appropriate consequences. They also described experiences in which women were blamed for inappropriate sexual comments and actions imposed on them by colleagues. They agreed that “more needs to be done” to adequately address these issues.

### Implicit biases

Unconscious biases and cultural expectations were themes expressed by participants in all career stages. This manifested as microaggressions and stereotype threats. The participants expressed being commonly misidentified as nurses and other ancillary staff and reported to often being referred to by their first name rather than title, in contrast to their male colleagues. One woman stated, “I was giving grand rounds and the surgeon that spoke after me, who is actually a very good friend of mine, thanked me by my first name and then the two men that had talked before me, as ‘doctor.’” Other women echoed this sentiment, indicating a common occurrence experienced by women in medicine.

Another common theme was the expectation of women to be modest and subdued in the workplace. This was exemplified by a statement made by a participant whose institution showed a video to women on professionalism and indicated that they should take down “offensive” pictures in their offices, such as being in a swimsuit. After viewing this video, the participant felt pressured to take down a picture of herself and her son on the beach. Other women noted feeling the need to “stay quiet” and “be agreeable.” One participant asserted this is likely a result of being taught from a young age that girls should be “likeable” and “nice.”

### Self-advocacy

These subtle cultural biases contribute to challenges women face asserting themselves in the workplace and maintaining appropriate boundaries with colleagues. Many women across career stages felt that colleagues often crossed boundaries with them when compared to their male counterparts. For example, one participant in the early-career group stated that the support staff often sought her out to vent about workplace concerns and would even say things that she described as “wildly inappropriate,” rather than seeking out the appropriate channels to report these issues.

Another participant in the mid-career group stated that the chair of her department often called at inappropriate times to discuss work matters, with no regard for her schedule. She felt that she could not push back or set boundaries and was expected to answer his calls at any time of day. Those in the late-career group described situations where they had to learn to advocate for themselves, particularly in situations where there were power differentials. One stated “when you're viewed as a very powerful person at the institution you get treated quite differently than if you're [earlier in your career].”

### Burnout/stress

Finally, women in all of the career groups described feelings of being “burned out” and “overwhelmed.” While this theme plagues both men and women in medicine, they identified unique struggles faced by women that may exacerbate burnout. The participants described how they bear most of the responsibility for childcare and housekeeping, in addition to their career responsibilities. One woman in the early career group expressed, “I feel the need to be extra successful or extra productive almost to show my male colleagues who question my maternity leaves or my motherhood, just that you know that's not a detriment to our group or to my success.”

Mid-career participants echoed these concerns, and also described burnout related to the challenges associated with managing patients with poor prognoses and their families. Those in the late-career group referred to stress related to the financial and retirement planning.

### Solutions for issues faced by women in medicine

Despite the many unique challenges faced, participants remained optimistic about the future of women in medicine. They proposed many solutions to the aforementioned issues. The major subthemes noted include (1) fostering supportive communities, (2) encouraging personal and professional development, and (3) establishing system wide policy changes that promote equity.

### Fostering supportive communities

All three focus groups discussed the importance of supporting women through work communities and support groups, where they could acknowledge common challenges, validate each other's experiences, share with male colleagues the unique challenges faced by women and have them as advocates, and focus on successful work-life integration strategies. A woman in the late-career group believes that “support systems [in medicine] are still behind the times.” She felt that efforts should be made to support “young women having babies or growing their families.” Participants in all groups expressed the affirmation received by their work communities, attending conferences that aim to empower women physicians, and having a space to discuss common struggles.

One woman attended a conference for women in medicine and learned that no perfect leaders exist, which helped her to feel empowered to seek leadership positions. In response, another participant described a colleague who attended a conference which resulted in her taking actions that lead to a promotion within a year. In addition to women supporting each other, they also described the importance of having men support women in the workplace. The participants gave many examples of men speaking out against overt and subtle discriminatory remarks made toward women in medicine. The women felt very supported and validated by having the support of their male colleagues.

### Encouraging personal and professional development

Participants found that building skills in self advocacy and negotiation, advocating for others through mentorship and other means, and taking time to care for oneself may help women more tactfully address the issues faced by women in medicine. “Take your power back and be your best advocate [for yourself] and for other people,” exclaimed one of the participants. All three groups expressed similar sentiments, but the early-career group specifically focused on building self-advocacy and negotiation skills. An early career female physician described her experience working with two male colleagues who bond over their similar interests, often shutting her out of decisions. She learned to assert herself as a co-decision-maker to try and advance more successfully.

Another early career physician has found that a different interpersonal dynamic is created by simply addressing herself as “Dr. [last name]” rather than by her first name. A woman in the late-career group hoped young learners and staff will feel empowered to “snap back” when they feel “dissed” by those in authoritative positions. Others in the late-career group were excited about the progress that is being made. “It's an exciting time to be a woman in medicine because of the energy the younger women and the change that they are bringing to the profession.”

### System-wide policy changes

The participants proposed policy and organizational changes that promote equity and empower women to assume leadership roles to systematically change the cultural undercurrents that hinder women's success. One of the participants works in a male predominated orthopedic surgery group that has sought out hiring more women. All of the groups acknowledged the benefit of having more women in medicine, including the purported cultural changes related to how women are viewed in medicine that will ensue. One woman suggested that having more women in medicine, specifically in “high leadership” positions, will result in “less sexual harassment because there will be less of a power differential, less men at the top.”

Women in the late-career group also suggested requiring institutional transparency regarding reported grievances. For example, a woman stated that organizations should report the number of sexual harassment complaints and the outcomes of those complaints. This would hold institutions accountable for appropriately handling workplace malfeasance. Another participant in her mid-career acknowledged the benefits of transparency, in that she feels more supported in her institution given its “openness about gender issues.”

### Definitions of success

The participants' definitions of success were categorized into two subthemes: (1) personal success, which they defined as happiness, feeling that they are serving a higher purpose, being well rounded, and establishing good work life balance and (2) leaving a legacy: supporting future generations, changing the culture, being a good leader, and representing women well.

### Personal success

The early-career group focused on finding fulfillment in defining their careers. A young pediatric oncologist divided her definition of success into clinical successes and academic successes. Clinical success being, for example, a patient she cured of cancer who was able to celebrate a wedding. Academic successes, for example, would be attaining higher leadership roles to ultimately mentor future young physicians.

The mid- and late-career participants felt that success was defined as happiness and being a well-rounded individual. A participant in her mid-career summed this up by stating, “You're not going to be the best wife. You're not going to be the best doctor. You're not going to be the best, but you can be pretty darn good at all of them.” A woman in the late-career group wished she had seen medicine as a part of her life, rather than her entire life, and achieved a better work/life balance earlier in her career, a sentiment shared by many of the participants.

### Leaving a legacy

Leaving a legacy is an idea discussed at length in all group discussions. They discussed the importance of setting an example for their sons and daughters, young female patients, and marginalized groups. “I feel being in our role has a positive influence on young girls and teenagers who are starting to think more about what they maybe want to do with their lives professionally.” Participants in the mid- and late-career group discussed the importance of recognizing the “big picture” and incorporating lessons learned from the past. They spoke about their previous situations giving momentum to understand the ways their careers can be shaped, situations to avoid, and how to help others in earlier career stages.

Those in the late-career group elaborated on the clear imbalances experienced within the system, but were enthusiastic to provide guidance and mentorship as they noticed opportunities for growth for women physicians as leaders in medicine. The participants were generally optimistic on the career trajectory of women in medicine and sought to leave their legacy through their relationships with others, as well as their own personal and professional development.

## Discussion/Limitations

Over the past several decades, many systemic and policy advancements have been implemented to address gender discrimination in medicine. For example, the Civil Rights Act of 1964 criminalized the failure or refusal to hire or discharge any individual, or otherwise to discriminate against any individual with respect to compensation, terms, conditions, or privileges of employment, because of an individual's race, color, religion, sex, or national origin.^33^ This act was later amended to include a Pregnancy Discrimination Act in 1974, which prohibited discrimination based on pregnancy when it comes to any term or condition of employment.^34,35^ More recent attention has been made to the gender pay gap, and institutions have begun developing policies to close these gaps.^25^

Early career women in this study experienced more implicit biases in their workplace interaction. Their self-advocacy was in the form of establishing a solid foundation for their career development and leadership roles. They expressed the pressure they felt to work harder than their male colleagues to achieve the same level of success.

Although many policies addressing gender inequity were made before the beginning of many of this study participants' careers, the mid- and late-career groups discussed a variety of examples where they experienced overt discrimination, many of which directly violated the above acts. Mid-career women expressed more instances of overt discrimination. Their self-advocacy centered on maintaining balance in their home and work life. They mentioned managing patients with increasing complexity of diagnoses and care needs. Early-career participants, on the contrary, did not discuss such experiences, suggesting that blatant discrimination in the workplace may have diminished or the early-career participants may not have recognized it, given how pervasive it is in the work culture.

However, the participants in all groups equally discussed their experiences with implicit biases. A systematic review analyzing gender inequities among female plastic surgeons supports this finding, citing several studies that demonstrate a shift over the last 25 years from explicit biases against women to more implicit biases.^36^

Late-career women also discussed overt discrimination at the policy and structural level. They recognized the continuous struggle and cycle of imbalance in organizational structure, however, their experience, in part, enabled them to also find opportunities to develop their careers and play to their professional strengths. Their financial planning and retirement were among the more significant burdens discussed.

While there were differences in the challenges of women at various stages in their career, it is clear that women continue to experience inequities in various aspects of their overall careers, including in compensation, career advancement, and overall career satisfaction.^2–13^ The method of discrimination may be evolving, but unfortunately the outcomes remain the same. Policies addressing overt discriminatory practices in the workforce are insufficient to change inequities faced by women in medicine. Until deeply ingrained unconscious biases are recognized and addressed, women will continue to face injustices in medicine.

Various potential solutions to address these inequities were identified under the categories of supportive communities, personal and professional development, and system-wide policy changes. The participants emphasized developing peer support systems, which would allow women physicians to develop a network of support, to create a space to discuss common struggles, to build one another up and collectively support the advancement of women in medicine. They also noted the importance of garnering validation and support from their male colleagues.

Regarding personal and professional development, a key factor that emerged from the data was the importance of mentorship, particularly within the early career group, the development of self-advocacy and negotiation skills, and coping with problems related to gender discrimination and disparity. Finally, the groups identified a need for systems of accountability at various levels of leadership to implement structural changes that mitigate gender discrimination and ensure the fair advancement of women in medicine.

This study was not without limitations. First, the participants were attendees at a conference with an advertised aim to “empower women and men in medicine with the skills and resources to remove barriers and bias…” and therefore likely attracted attendees who either experienced barriers and biases in their practice and/or who felt strongly about the topics.

In addition, the guidance of the discussion was purposely limited, and therefore the fact that a topic was not mentioned, does not necessarily indicate that participants did not have that particular experience. Finally, participants were largely white and partnered with children, and it is not known whether the majority were in an academic setting or private practice; therefore, their experiences may not be representative of the full range of intersectionality present among women physicians in a variety of settings.

## Conclusions/Implications

Findings from focus group interviews with women physicians identified that, across career stages, they tend to face systemic barriers to success, implicit biases, a need for self-advocacy, and symptoms of burnout and stress. Systemic barriers to success were more prevalent among the mid- and late-career groups and included policies and practices that contribute to inequities for female physicians. Implicit biases were also identified across all three career stages and encompassed cultural expectations of women that tend to manifest as microaggressions and stereotype threats. Participants in the mid- and late-career groups expressed feeling burned out and overwhelmed due to a combination of career and domestic expectations leading to issues with work-life integration.

Several solutions to the issues were identified, including fostering a supportive community of women in medicine through collegial support groups, and through garnering support from male colleagues. Other potential solutions included encouraging personal and professional development (*i.e.*, self-advocacy, negotiation skills, and mentorship opportunities) and system-wide policy changes that promote equity and empower women to assume leadership roles. Finally, conference attendees indicated their measures of success, which included both personal success and leaving a legacy.

Despite policy advancements and a social evolution away from overt discriminatory practices, women in medicine continue to experience inequities. The methods of discrimination may be evolving; however, outcomes that disadvantage women remain the same. These biases and inequities must be addressed and reformed to empower the fair advancement of women in medicine.

## Abbreviation Used

GRIT: Growth, Resilience, Inspiration, and Tenacity
